# Appointment

**Published:** 1873-11

**Authors:** 


Appointment. Dr. Alfred T. Livingston, a graduate of the Buffalo Medical College of the class of 1873, and who has favored the Journal with a few contributions has been appointed one of the Assistant Physicians at the State Insane Asylum at Utica. We wish Dr. Livingston much pleasure in his new field of labor, and congratulate his associates upon securing so intelligent and agreeable a companion.

